# Identifying the Potential Roles of PBX4 in Human Cancers Based on Integrative Analysis

**DOI:** 10.3390/biom12060822

**Published:** 2022-06-13

**Authors:** Yipeng Song, Rongna Ma

**Affiliations:** 1 School of Law and Criminal Justice, East China University of Political Science and Law, No. 555 Longyuan Road, Songjiang University Town, Shanghai 201620, China; songyipeng@ecupl.edu.cn; 2 Shanghai Institute of Blood Transfusion, Shanghai Blood Center, No. 1191 Hongqiao Road, Shanghai 200051, China

**Keywords:** PBX4, cancer, integrative analysis, mutation, DNA methylation, immune infiltrating

## Abstract

PBX4 belongs to the pre-B-cell leukemia homeobox (PBX) transcription factors family and acts as a transcriptional cofactor of HOX proteins participating in several pathophysiological processes. Recent studies have revealed that the dysregulation of PBX4 is closely related to multiple diseases, especially cancers. However, the research on PBX4’s potential roles in 33 cancers from the Cancer Genome Atlas (TCGA) is still insufficient. Therefore, we performed a comprehensive pan-cancer analysis to explore the roles of PBX4with multiple public databases. Our results showed that PBX4 was differentially expressed in 17 types of human cancer and significantly correlated to the pathological stage, tumor grade, and immune and molecular subtypes. We used the Kaplan–Meier plotter and PrognoScan databases to find the significant associations between PBX4 expression and prognostic values of multiple cancers. It was also found that PBX4 expression was statistically related to mutation status, DNA methylation, immune infiltration, drug sensitivity, and immune checkpoint blockade (ICB) therapy. Additionally, we found that PBX4 was involved in different functional states of multiple cancers from the single-cell resolution perspective. Enrichment analysis results showed that PBX4-related genes were enriched in the cell cycle process, MAPK cascade, ncRNA metabolic process, positive regulation of GTPase activity, and regulation of lipase activity and mainly participated in the pathways of cholesterol metabolism, base excision repair, herpes simplex virus 1 infection, transcriptional misregulation in cancer, and Epstein–Barr virus infection. Altogether, our integrative analysis could help in better understanding the potential roles of PBX4 in different human cancers.

## 1. Introduction

Given the complex pathogenesis of cancers, multiple omics analysis (i.e., genomic, transcript, and protein levels) of cancer-related genes can help researchers to further understand their roles in the occurrence and development of tumors [1,2,3]. With the development of biological detection and bioinformatics technology, the growth in cancer databases, such as TCGA, Gene Expression Omnibus (GEO), and Human Protein Atlas (HPA), makes it possible to perform integrative pan-cancer analysis of genes of interest [4,5,6].

PBX4 is a member of the PBX transcription factors family, which plays vital roles in the development of multiple organs [7,8,9], embryonic development [10], myocardial differentiation [11], neural crest cell migration [12], and spermatogenesis [13]. In recent years, an increasing number of studies have uncovered that PBX4 dysregulation is closely related to various diseases, especially cancers. The dyslipidemia of PBX4 loci (rs16996148) was associated with cardiovascular disease [14]. Vsedova et al. found that PBX4 was related to metabolic syndrome [15]. Abnormal expression of PBX4 occurred in Hodgkin lymphoma patients [16] and acute lymphoblastic leukemia [17]. Palles et al. found that PBX4 participated in the development of esophageal cancer [18]. Martinou et al. revealed that upregulation of PBX4 might shorten the survival time of patients with colorectal cancer [19]. However, these studies of PBX4 were restricted to a single cancer. The multiple omics alterations of the genes in different types of human cancer may have significant differences [20,21].

**Figure 1:**
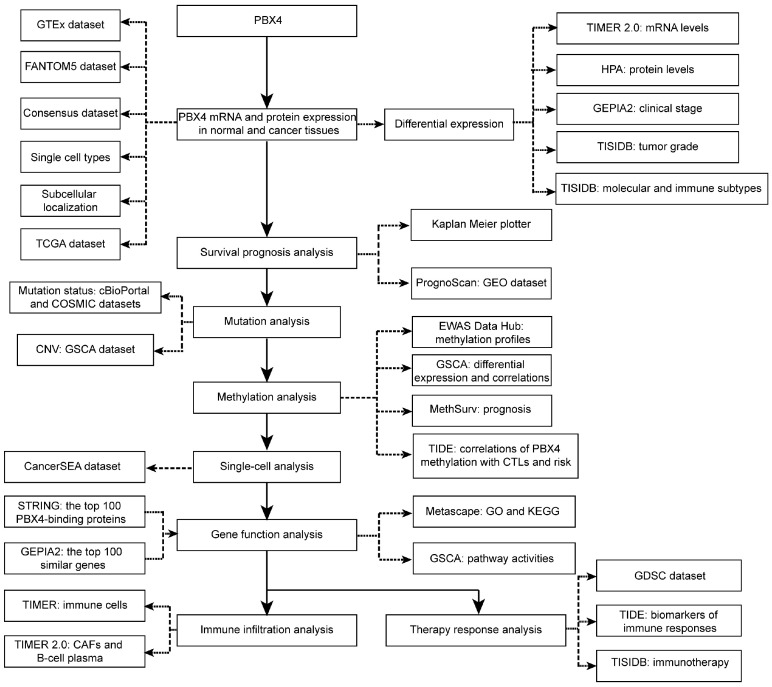
The study design of our work.

Therefore, for the first time, we performed integrative analysis to provide a comprehensive understanding of the roles of PBX4 in different human cancer types (Figure 1). We explored differential expression of PBX4 across TCGA cancers. Then, we investigated survival prognosis, molecular and immune subtypes, mutation status, DNA methylation, single-cell functional states, and relevant biological process and pathways. Finally, we performed immune infiltration analysis and therapy response analysis. Our results reveal the potential roles of PBX4 in the occurrence, development, and treatment of multiple human cancers. PBX4 could serve as a biomarker for cancer detection, prognosis, and immunotherapy.

## 2. Materials and Methods

### 2.1. Analysis of PBX4 Gene Expression in Cancer and Normal Tissues

The PBX4 mRNA expression in normal and cancer tissues was explored using the Human Protein Atlas (HPA) database (http://www.proteinatlas.org/, accessed on 15 April 2022) [6]. We used the ‘Tissue’ section to explore PBX4 RNA expression in the Genotype-Tissue Expression (GTEx, RNA-seq data from 36 tissue types), FANTOM5 (RNA expression data from 60 tissues), and Consensus (55 tissue types combining the HPA and GTEx RNA-seq data) datasets and PBX4 protein expression in the HPA datasets (protein data from 44 tissues). ‘Single cell type’ was used to study the single-cell RNA levels of PBX4 from all single cell types. We used the ‘Subcell’ section to determine the subcellular location of PBX4 with indirect immunofluorescence microscopy. The ‘Pathology’ section was used to explore the RNA expression of PBX4 in TCGA dataset and protein expression of PBX4 in multiple cancer tissues. Tumor Immune Estimation Resource (TIMER) is a comprehensive and intuitive web resource for the analysis of differential expression and immune infiltrates. We used the ‘Gene_DE’ module of TIMER 2.0 (http://timer.cistrome.org/, accessed on 15 April 2022) to investigate the PBX4 differential expression between cancers and paired normal tissues in all TCGA cancers [22] (the abbreviations and corresponding full names of various cancers are shown in Table 1). GEPIA2 (http://gepia2.cancer-pku.cn/, accessed on 15 April 2022, the RNA-seq data of 8587 normal samples and 9736 tumors in the GTEx and TCGA datasets) was used to show expression violin plots of the pathological stage [23]. Based on multiple heterogeneous datasets, the Tumor and Immune System Interaction Database (TISIDB) (http://cis.hku.hk/TISIDB/index.php, accessed on 15 April 2022) is an interactive web portal for the analysis of tumors and the immune system [24]. Moreover, we used the ‘Subtype’ module of TISIDB to analyze the correlations between pbx4 expression and molecular and immune subtypes in different types of human cancer.

### 2.2. Survival Prognosis Analysis

The Kaplan–Meier plotter (https://kmplot.com/analysis/, accessed on 15 April 2022) is an intuitive web tool for survival analysis of 30,000 genes in 25,000+ samples from 21 types of human cancer (Gene Expression Omnibus (GEO), European Genome-phenome Archive (EGA), and TCGA). By dividing the patients into high-expression and low-expression PBX4 groups, we used the Kaplan–Meier plotter to assess the associations between PBX4 expression and overall survival (OS) and relapse-free survival (RFS) across cancers [25]. Furthermore, we used PrognoScan (http://www.prognoscan.org/, accessed on 15 April 2022) to further verify our findings [26].

### 2.3. Mutation Status Analysis

With 10967 samples from TCGA data, the ‘Quick Select’ section of cBioPortal (https://www.cbioportal.org/, accessed on 15 April 2022) was used to explore the genetic alteration status of PBX4 in all TCGA cancers [27,28]. The ‘Cancer Types Summary’ module was used to analyze the overview of PBX4 mutation status, and the ‘Mutations’ module was used to show PBX4 mutation sites and corresponding 3D structures. Moreover, the ‘Comparison/Survival’ module was used to explore the influence of PBX4 mutation status on pan-cancer survival prognosis. The distribution of putative copy number alterations (CNA) of PBX4 expression from the GISTIC database was shown in the ‘Plots’ module. COSMIC (https://cancer.sanger.ac.uk/cosmic, accessed on 15 April 2022, over 37,000 genomes) was applied to verify the pan-cancer PBX4 mutation features [29]. By integrating over 10,000 multi-dimensional genomic data of 33 cancer types in TCGA and over 750 small molecule drugs in CTRP and GDSC, Gene Set Cancer Analysis (GSCA) (http://bioinfo.life.hust.edu.cn/GSCA/#/, accessed on 15 April 2022) can be used to perform CNV analysis and could search valuable small drugs [30]. We used GSCA to analyze the correlation between PBX4 expression and CNV in different cancers and the effect of PBX4 CNV on the pan-cancer survival prognosis.

### 2.4. Analysis of PBX4 DNA Methylation

EWAS Data Hub (https://ngdc.cncb.ac.cn/ewas/datahub/index, accessed on 15 April 2022) is a data hub for the analysis of DNA methylation in 115,852 samples of 528 diseases. We used EWAS Data Hub to explore DNA methylation profiles of PBX4 in different human tissues [31,32]. GSCA was used to find the difference of PBX4 methylation in cancer tissues and paired normal tissues and the correlations between PBX4 DNA methylation levels and PBX4 expression. MethSurv (https://biit.cs.ut.ee/methsurv/, accessed on 15 April 2022) is an intuitive web tool for multivariable survival analysis of DNA methylation from 25 human cancers and 7358 patients [33] and was used to analyze the influence of PBX4 methylation on survival prognosis of different TCGA cancers. Tumor Immune Dysfunction and Exclusion (TIDE) (http://tide.dfci.harvard.edu/, accessed on 15 April 2022, with over 33,000 samples of 188 tumor cohorts and 998 tumors from 12 ICB studies) is an intuitive and interactive web platform for the study of gene functions in modulating tumor immunity and biomarkers in ICB responses [34]. The ‘Query Gene’ section of TIDE was used to explore the relevance of PBX4 methylation and cytotoxic T-cell levels and the risk of various cancers.

### 2.5. Single-Cell Analysis

Cancer Single-Cell State Atlas (CancerSEA) (http://biocc.hrbmu.edu.cn/CancerSEA/, accessed on 15 April 2022) provides an intuitive online resource for researchers to perform single-cell analysis of 14 functional states in 41,900 cancer single cells from 25 types of human cancer [35]. We used the ‘Search PCG/lncRNA for the associated functional states’ module to study the functional states of PBX4 in various cancers.

### 2.6. PBX4-Related Partner Function Analysis

STRING (https://string-db.org/, accessed on 15 April 2022, 14,094 organisms, 67.6 million proteins, and over 2 billion interactions) was used to obtain PBX4-binding proteins and construct the corresponding protein–protein interaction network [36]. The ‘Similar Genes Detection’ of GEPIA2 was applied to search for 100 genes with similar pan-cancer expression patterns of PBX4. Then, we used Venny 2.1 (https://bioinfogp.cnb.csic.es/tools/venny/index.html, accessed on 15 April 2022) to perform an intersection analysis of the above two groups. Gene Ontology (GO) and Kyoto Encyclopedia of Genes and Genomes (KEGG) enrichment analysis of these PBX4-related partners was analyzed using Metascape (http://metascape.org/, accessed on 15 April 2022) [37]. Moreover, we used the ‘Single gene level analysis’ module of GSCA to investigate the effect of PBX4 expression on various pathway activities in different TCGA cancers.

### 2.7. Immune Infiltration Analysis

We used the ‘Mutation’ module of TIMER 2.0 to analyze the correlation between PBX4 expression and infiltration of cancer-associated fibroblasts and B-cell plasma across TCGA cancers. Then, TIMER (https://cistrome.shinyapps.io/timer/, accessed on 15 April 2022) was applied to study the associations between PBX4 expression and B cells, CD8+ T cells, CD4+ T cells, and dendritic cells in multiple human cancers [38].

### 2.8. Therapy Response Analysis

The ‘Drug Sensitivity’ module of GSCA was used to explore the drug sensitivity analysis of PBX4 expression from the GDSC database. We used the ‘Biomarker Evaluation’ module of TIDE to find the difference between PBX4 and existing biomarkers of cancer immune evasion in the immune checkpoint blockade. Additionally, TISIDB was used to evaluate the relationship between PBX4 expression with cytotoxic T-cell levels and the influence of PBX4 expression on the survival prognosis of immunotherapeutic responses.

## 3. Results

### 3.1. PBX4 Expression in Normal Human Tissues and Its Localization

**Figure 2:**
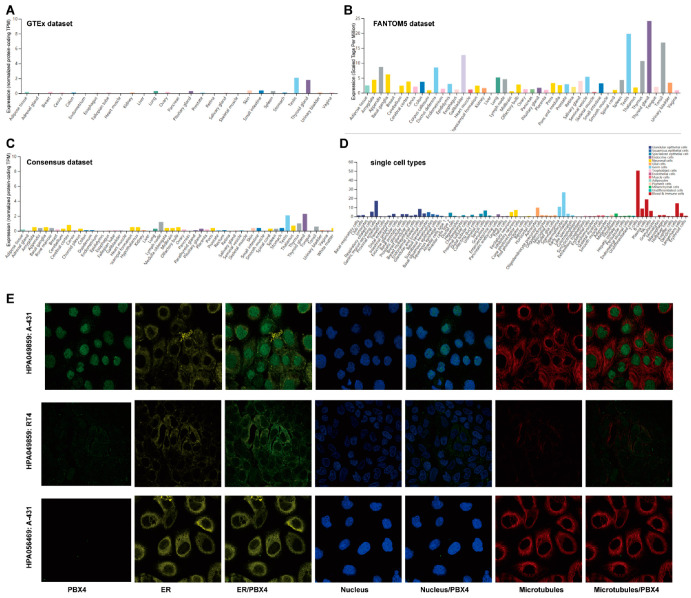
The expression levels of PBX4 in normal human tissues and its localization. (**A**) The mRNA expression of PBX4 in different human tissues from the GTEx dataset. (**B**) The mRNA expression of PBX4 in different human tissues from the FANTOM5 dataset. (**C**) The mRNA expression of PBX4 in different human tissues from the Consensus dataset. (**D**) Overview of PBX4 expression from all single cell types. (**E**) Immunofluorescence staining of the subcellular localization of PBX4 in the endoplasmic reticulum (ER), nucleus, and microtubules of A-431 (#HPA056469), RT4 (#HPA049859), and A-431 (#HPA049859) obtained from the HPA database.

We used the GTEx, FANTOM5, and Consensus datasets to explore the distribution of PBX4 expression in normal human tissues. As shown in Figure 2A, the mRNA expression of PBX4 was primarily expressed in the thyroid gland, testis, small intestine, skin, pituitary gland, and lung tissues from the GTEx dataset. Moreover, we found that the major tissues of PBX4 expression were the thyroid gland, testis, gallbladder, tonsil, thymus, appendix, and ductus deferens in the FANTOM5 dataset (Figure 2B). Interestingly, the results of the Consensus dataset also revealed that PBX4 was primarily located in the thyroid gland, testis, lymph node, thymus, and cerebral cortex tissues (Figure 2C). All three datasets showed that PBX4 was more highly expressed in the thyroid gland and testis than in other tissues. The results of the RNA single-cell-type specificity indicated that PBX4 was mainly expressed in the blood and immune cells, germ cells, glandular epithelial cells, and adipocytes (Figure 2D). To acquire experimental evidence on the subcellular localization of PBX4, we used HPA to perform an immunofluorescence analysis of the ER and microtubules of A-431 (#HPA056469), RT4 (#HPA049859), and A-431 (#HPA049859) cells. The results showed that PBX4 was mainly localized in vesicles and nucleoplasm (Figure 2E).

### 3.2. PBX4 Expression Levels in Various Human Cancers

**Figure 3:**
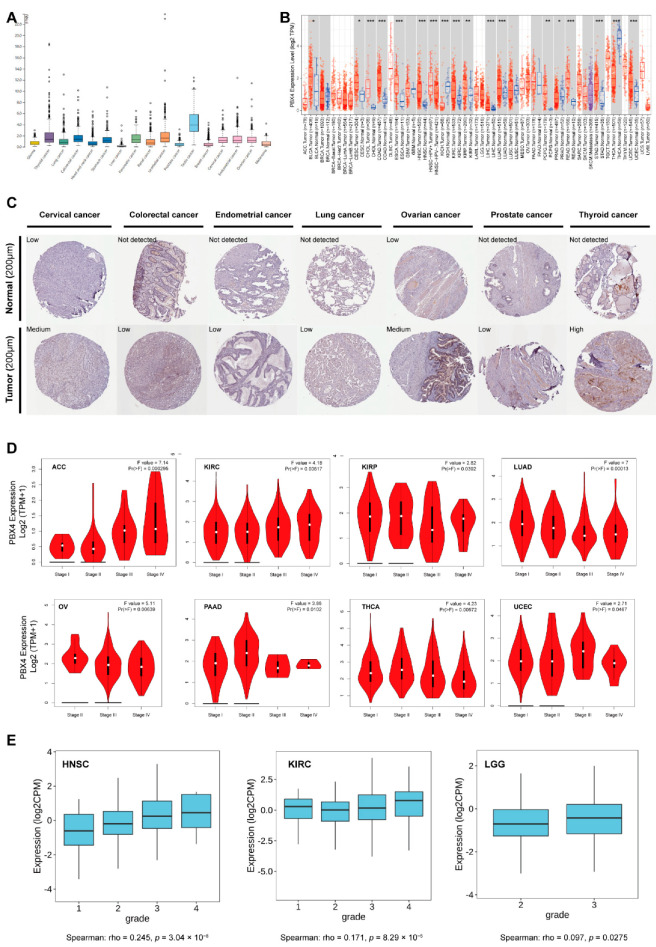
PBX4 expression levels in various human cancers. (**A**) The PBX4 mRNA expression in cancer tissues from TCGA database. (FPKM: fragments per kilobase of exon per million reads). (**B**) Differential expression of PBX4 in TCGA cancers and normal tissues of TIMER 2.0 (* *p* < 0.05, ** *p* < 0.01, *** *p* < 0.001). (**C**) The immunohistochemistry images of PBX4 in different tumor (down) and paired normal (up) tissues. (**D**) The associations between PBX4 expression and clinical stage in ACC, KIRC, KIRP, LUAD, OV, PAAD, THCA, and UCEC. (**E**) The associations between PBX4 expression and tumor grade in HNSC, KIRC, and LGG.

As shown in Figure 3A, we found that PBX4 was highly expressed in the tissues of testis cancer, thyroid cancer, colorectal cancer, urothelial cancer, stomach cancer, pancreatic cancer, cervical cancer, endometrial cancer, and ovarian cancer from TCGA database. Moreover, we used TIMER 2.0 to explore the differential expression of PBX4 between tumor tissues and adjacent normal tissues. The results showed that PBX4 was abnormally expressed in most human cancer types (Figure 3B). Specifically, PBX4 was significantly overexpressed in 14 cancer types, namely BLCA, CESC, CHOL, COAD, ESCA, HNSC, KIRC, KIRP, LIHC, LUAD, PCPG, READ, STAD, and UCEC, and significantly underexpressed in PRAD, THCA, and KICH. Moreover, we investigated PBX4 protein expression in various cancers. The results showed that cervical cancer, colorectal cancer, endometrial cancer, lung cancer, ovarian cancer, prostate cancer, and thyroid cancer had medium or low immunohistochemistry staining, while their paired normal tissues had no detection or low staining (Figure 3C).

Furthermore, the results of GEPIA2 revealed that significant associations between PBX4 expression and clinical stage were found in ACC, KIRC, KIRP, LUAD, OV, PAAD, THCA, and UCEC (Figure 3D). For ACC and KIRC, as the individual clinical stage progressed, the PBX4 expression increased, and the highest mRNA level was in stage IV. On the contrary, PBX4 expression decreased as the clinical stage of LUAD and OV progressed, and the lowest mRNA level was in stage IV. With the TISIDB database, we found that PBX4 expression was associated with a tumor grade of HNSC (*p* = 3.04 × 10^−8^), KIRC (*p* = 8.29 × 10^−5^), and LGG (*p* = 0.0275) (Figure 3E). As the tumors’ grades progressed, their PBX4 expression increased. For instance, the PBX4 levels of HNSC were highly expressed in grade 4, moderately expressed in grades 2 and 3, and expressed at a low level in grade 1. These results suggest that PBX4 might be a critical gene that promotes the progression of multiple cancers.

### 3.3. Correlations between PBX4 Expression and Molecular Subtypes and Immune Subtypes of Different Cancers

**Figure 4:**
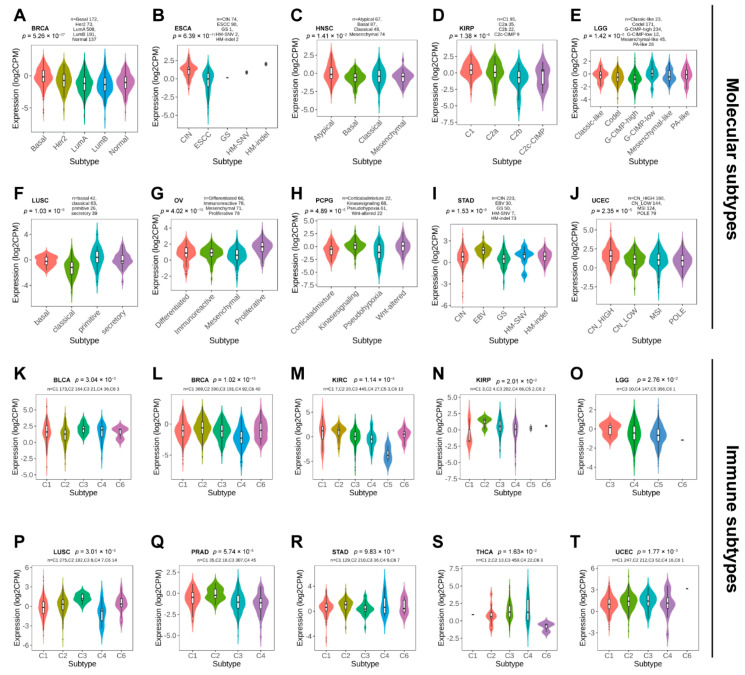
The PBX4 expression in molecular subtypes and immune subtypes across human cancers. Correlations between PBX4 expression and molecular subtypes of (**A**) BRCA, (**B**) ESCA, (**C**) HNSC, (**D**) KIRP, (**E**) LGG, (**F**) LUSC, (**G**) OV, (**H**) PCPG, (**I**) STAD, and (**J**) UCEC. Correlations between PBX4 expression and immune subtypes of (**K**) BLCA, (**L**) BRCA, (**M**) KIRC, (**N**) KIRP, (**O**) LGG, (**P**) LUSC, (**Q**) PRAD, (**R**) STAD, (**S**) THCA, and (**T**) UCEC. C1 (wound healing); C2 (IFN-gamma dominant); C3 (inflammatory); C4 (lymphocyte depleted); C5 (immunologically quiet); C6 (TGF-beta dominant).

Next, we used TISDB to explore the correlations between PBX4 expression and molecular subtypes and molecular subtypes across TCGA cancers. Violin plots demonstrated PBX4 expression was statistically associated with different molecular subtypes of BRCA, ESCA, HNSC, KIRP, LGG, LUSC, OV, PCPG, STAD, and UCEC (Figure 4A–J). Specifically, PBX4 was highly expressed in the Basal subtype and expressed at a low level in the LumB subtype of BRCA. PBX4 had the highest expression in the HM-indel subtype of ESCA, the Atypical subtype of HNSC, the Atypical C1 of KIRP, and the G-CIMP-low subtype of LGG. For LUSC, PBX4 had the highest expression in the primitive subtype than in other subtypes. PBX4 was highly expressed in the proliferative subtype of OV, the Wnt-altered subtype of PCPG, and the EBV subtype of STAD. For UCEC, the highest and lowest expression of PBX4 was in the CN-HIGH and POLE subtypes, respectively.

Additionally, we found that there were significant correlations between PBX4 expression and six immune subtypes (i.e., wound healing, IFN-gamma dominant, inflammatory, lymphocyte depleted, immunologically quiet, and TGF-beta dominant) of 10 cancer types, namely BLCA, BRCA, KIRC, KIRP, LGG, LUSC, PRAD, STAD, THCA, and UCEC (Figure 4K–T). For KIRP, PRAD, and STAD, PBX4 had a higher expression in the IFN-gamma dominant immune subtype than in other subtypes. PBX4 had the highest expression in the wound healing subtype of BLCA and KIRC and the inflammatory subtype of LGG, LUSC, and THCA. For BRCA and UCEC, the highest expression of PBX4 was found in the TGF-b dominant subtype. In contrast, PBX4 had the lowest expression in the lymphocyte depleted subtype of BRCA, LUSC, and PRAD. The lowest levels of PBX4 in BLCA and KIRC was found in the IFN-gamma dominant and immunologically quiet subtypes, respectively. For KIRP and UCEC, the lowest expression occurred in the wound healing subtype. PBX4 was expressed at a low level in the TGF-b dominant subtype of LGG and THCA.

### 3.4. Prognostic Analysis of PBX4 across Human Cancers

**Figure 5:**
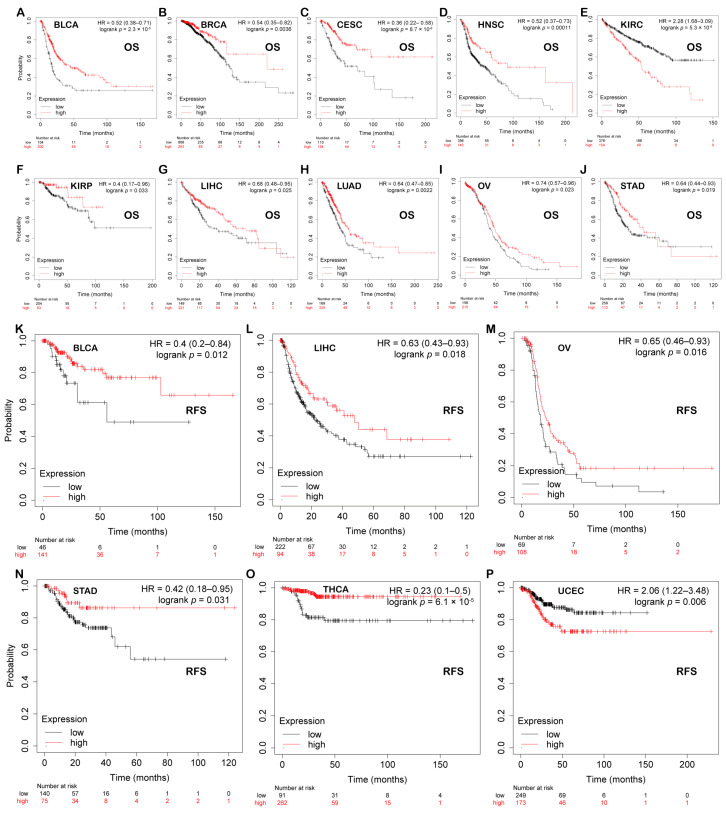
Prognostic value of PBX4 across human cancers with Kaplan–Meier plotter. Significant correlations between high-expression and low-expression groups of PBX4 and OS in (**A**) BLCA, (**B**) BRCA, (**C**) CESC, (**D**) HNSC, (**E**) KIRC, (**F**) KIRP, (**G**) LIHC, (**H**) LUAD, (**I**) OV, and (**J**) STAD. Significant correlations between high-expression and low-expression groups of PBX4 and RFS in (**K**) BLCA, (**L**) LIHC, (**M**) OV, (**N**) STAD, (**O**) THCA, and (**P**) UCEC.

**Figure 6:**
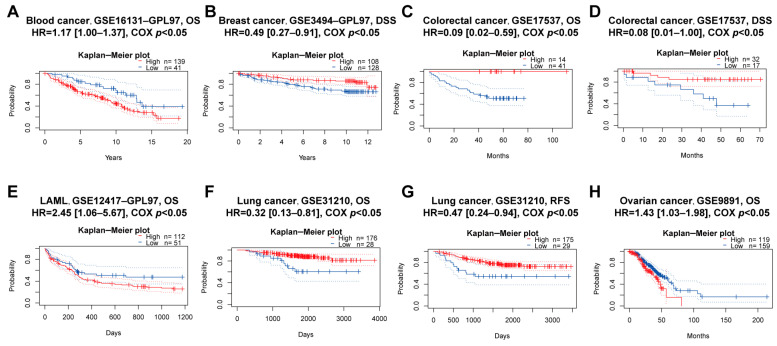
Prognostic value of PBX4 across human cancers with PrognoScan. Significant correlations between high-expression and low-expression groups of PBX4 and (**A**) OS of blood cancer, (**B**) DSS of breast cancer, (**C**) OS and (**D**) DSS of colorectal cancer, (**E**) OS of LAML, (**F**) OS and (**G**) RFS of lung cancer, and (**H**) OS of ovarian cancer.

We explored the associations between PBX4 expression and OS and RFS in different cancer types by using the Kaplan–Meier plotter. As shown in Figure 5A–J, it was found that the high-expression group of PBX4 had a favorable prognosis of OS in BLCA (*p* = 2.3 × 10^−5^), BRCA (*p* = 0.0036), CESC (*p* = 8.7 × 10^−6^), HNSC (*p* = 0.00011), KIRP (*p* = 0.033), LIHC (*p* = 0.025), LUAD (*p* = 0.0022), OV (*p* = 0.023), and STAD (*p* = 0.019) but a poor prognosis in KIRC (*p* = 5.3 × 10^−8^). As for RFS, low expression of PBX4 indicated a poor prognosis in five cancer types (BLCA, LIHC, OV, STAD, and THCA) but a good prognosis in UCEC (Figure 5K–P). Next, we further validated the prognosis of different cancers by using the GEO dataset (Figure 6). The results revealed that high PBX4 expression indicated a good OS and disease-specific survival (DSS) of colorectal cancer in the GSE17537 cohort. High PBX4 expression suggested a good OS and RFS of lung cancer in the GSE31210 cohort. Breast cancer patients with low PBX4 expression implied a poor DSS (GSE3494-GPL97). High PBX4 expression was correlated with the poor OS in blood cancer (GSE16131-GPL97), ovarian cancer (GSE9891), and LAML (GSE12417-GPL97).

### 3.5. Mutation Analysis of PBX4 in Different TCGA Cancers

**Figure 7:**
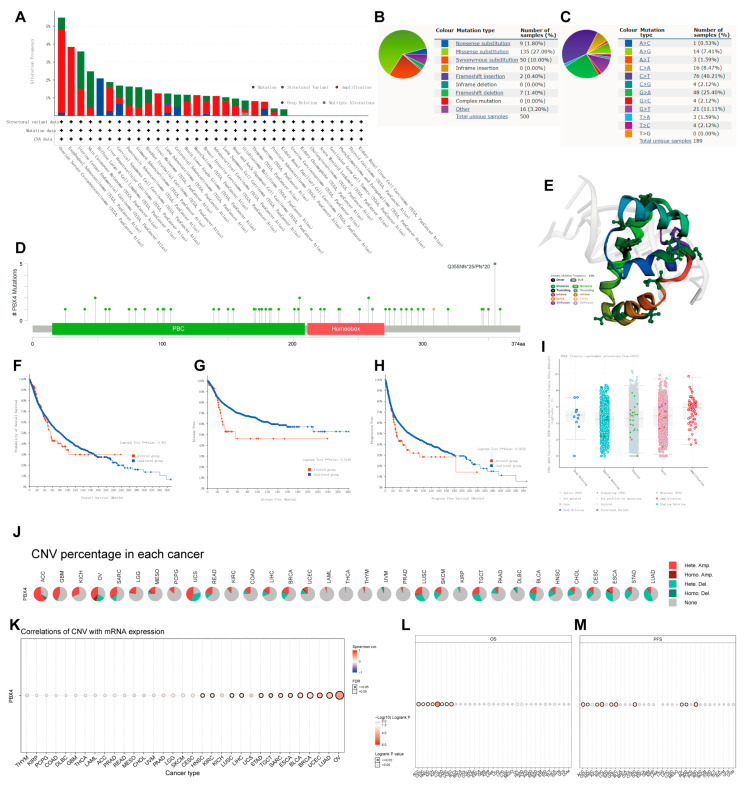
Mutation features of PBX4 in different human cancers. Overview of the mutation status of PBX4 across TCGA cancers by (**A**) cBioPortal and (**B**) COSMIC. (**C**) Summary of PBX4 substitutional mutation types by COSMIC. (**D**) Mutation sites and (**E**) corresponding 3D structures of PBX4 displayed by cBioPortal. The correlations between pan-cancer PBX4 mutation status and (**F**) OS, (**G**) DFS, and (**H**) PFS by cBioPortal. (**I**) The putative CNA of PBX4 from the GISTIC database. (**J**) Summary of PBX4’s CNV across TCGA cancers and (**K**) the correlation between PBX4 expression and CNV in different cancers by GSCA. The difference of (**L**) OS and (**M**) PFS between PBX4’s CNV and wide type in different cancers by GSCA.

To investigate the effects of mutation features on the occurrence and development of human cancers, we used cBioPortal and COSMIC to explore the pan-cancer PBX4 mutative status. As shown in Figure 7A, we found that the highest PBX4 alteration frequency occurred in OV, followed by ESCA, UCEC, SKCM, and DLBC. Furthermore, five types of PBX4 alteration (mutation, structural variant, amplification, deep deletion, and multiple alterations) were generally found in various TCGA cancers, in which ‘Amplification’ was the main type. From the PBX4 mutation sites (Figure 7D) and their corresponding 3D structures (Figure 7E), Q355Nfs*25/Pfs*20 was the most frequent mutation site and the ‘missense’ type was the primary type of pan-cancer mutation (49 missenses/58 mutations). Moreover, we found that the PBX4 expression of the ‘amplification’ type was higher than that of other types in putative CNA from the GISTIC database (Figure 7I). The COSMIC results also revealed that missense substitutions were the primary mutation type and took 27% of 500 PBX4 mutation samples. Moreover, PBX4 substitution mutations mostly occurred in C > T (40.21%) and G > A (25.40%). Next, we used cBioPortal to investigate the influence of PBX4 alterations on the pan-cancer survival prognosis. The results showed that PBX4 alterations could significantly shorten DFS (*p* = 0.0146) and PFS (*p* = 0.0232) but were not statistically associated with OS (*p* = 0.451) (Figure 7F–H). Furthermore, we explored the distribution of different types of PBX4’s CNV (Figure 7J) and the correlations between PBX4 expression and CNV (Figure 7K) in human cancers. The significant correlations between PBX4 expression and CNV could be found in HNSC, KIRC, LUSC, LIHC, STAD, TGCT, SARC, ESCA, BLCA, BRCA, UCEC, LUAD, and OV. In addition, the CNV of PBX4 was significantly correlated with poor OS of eight cancer types (ACC, CESC, HNSC, KICH, KIRC, LUAD, SARC, and UCEC) and poor PFS of 10 cancer types (ACC, CESC, KICH, KIRC, SARC, UCEC, COAD, OV, PAAD, and PRAD) (Figure 7L,M).

### 3.6. DNA Methylation Analysis of Pan-Cancer PBX4

**Figure 8:**
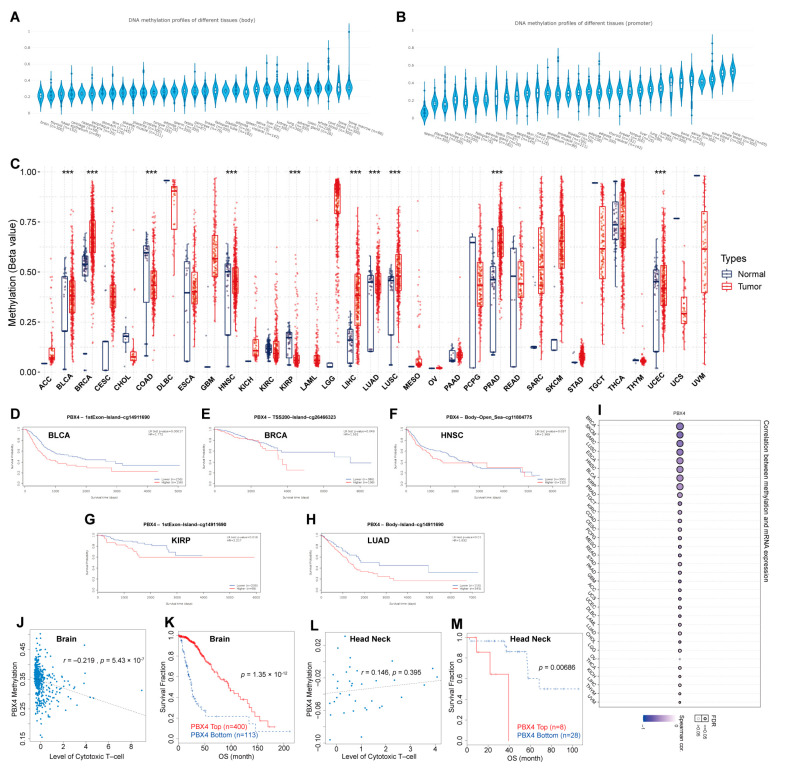
DNA methylation analysis of pan-cancer PBX4. PBX4 DNA methylation of (**A**) body and (**B**) promoter in human tissues. (**C**) The difference between PBX4 DNA methylation in cancer tissues and paired normal tissues (*** *p* < 0.001). The survival curves of PBX4 methylation for (**D**) CpG cg14911690 in BLCA, (**E**) CpG cg26466323 in BRCA, (**F**) CpG cg10738025 in HNSC, (**G**) CpG cg14911690 in KIRP, and (**H**) CpG cg11804775 in LUAD. (**I**) The correlations between methylation and expression of PBX4. The associations between the PBX4 methylation and cytotoxic T-cell level (CTL) in (**J**) brain and (**L**) head and neck. The influence of the PBX4 methylation on the OS curves of (**K**) brain and (**M**) head and neck.

Furthermore, we performed DNA methylation analysis of PBX4 across TCGA cancers. Firstly, we used EWAS Data Hub to find that PBX4 DNA methylation of body and promoter primarily occurred in bone, blood, lung, kidney, and liver tissues (Figure 8A,B). The GSCA results showed that hypermethylation of PBX4 occurred in BRCA, LIHC, LUAD, LUSC, PRAD, and UCEC, but hypomethylation occurred in BLCA, COAD, HNSC, and KIRP (Figure 8C). As shown in Figure 8I, PBX4 methylation was negatively correlated with PBX4 expression in different human cancers. We used MethSurv to analyze the influence of PBX4 methylation on the survival prognosis of various cancers. Kaplan–Meier plots revealed that PBX4 hypermethylation levels of BLCA, BRCA, HNSC, KIRP, and LUAD were significantly associated with poor prognosis (Figure 8D–H). In addition, we used TIDE to find that PBX4 methylation was negatively correlated with dysfunctional T-cell phenotypes, and hypermethylation of PBX4 implied a good prognosis (*p* = 1.35 × 10^−12^) in the brain. On the contrary, PBX4 methylation was positively correlated with dysfunctional T-cell phenotypes, and hypermethylation of PBX4 implied a poor prognosis (*p* = 0.00686) in the head and neck.

### 3.7. Single-Cell Functional Analysis of PBX4 across Human Cancers

**Figure 9:**
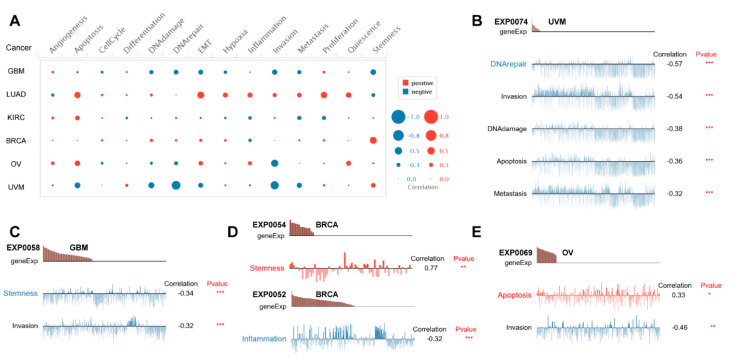
Single-cell functional analysis of PBX4 from the CancerSEA database. (**A**) Overview of PBX4 functional states in different human cancers. The functions were significantly correlated with PBX4 in (**B**) UVM, (**C**) GBM, (**D**) BRCA, and (**E**) OV. (* *p* < 0.05, ** *p* < 0.01, *** *p* < 0.001).

To further investigate potential roles of PBX4 in different cancers, we used CancerSEA to explore the functional heterogeneity of PBX4 at the single-cell resolution (Figure 9A). The results revealed that PBX4 expression was negatively related to DNArepair, invasion, DNAdamage, apoptosis, and metastasis in the EXP0074 dataset of UVM (all *p* < 0.001) (Figure 9B) and stemness (correlation = −0.34, *p* < 0.001) and invasion (correlation = −0.32, *p* < 0.001) in the EXP0058 dataset of GBM (Figure 9C). We found a significant relationship between the PBX4 expression of BRCA and stemness (correlation = 0.77) in the EXP0054 dataset and inflammation (correlation = −0.32) in the EXP0052 dataset (Figure 9D). Figure 9E showed that PBX4 expression in OV was positively associated with apoptosis (correlation = 0.33, *p* < 0.05) but negatively associated with invasion (correlation = −0.64, *p* < 0.01).

### 3.8. Functional Analysis of PBX4-Related Partners

**Figure 10:**
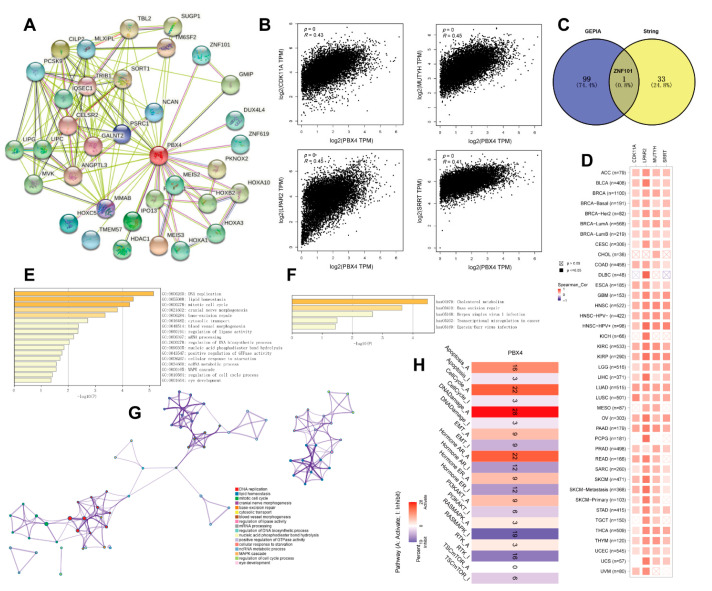
Functional analysis of genes and proteins relevant to PBX4. (**A**) STRING protein–protein interaction network of PBX4-binding proteins (nodes and edges represent proteins and protein–protein associations, respectively). (**B**) The top four genes (CDK11A, LPAR2, MUTYH, and SRRT) were most strongly correlated to PBX4 in TCGA database. (**C**) Intersection analysis of the PBX4-correlated genes and PBX4-binding proteins. (**D**) Heatmap of the expression correlations between PBX4 and CDK11A, LPAR2, MUTYH, and SRRT across TCGA cancers. (**E**) Biological process analysis of the PBX4-related genes (bar graph of enriched terms colored by *p*-values) and (**G**) the corresponding network of enriched terms (nodes of enriched terms colored by cluster ID). (**F**) KEGG pathway analysis of PBX4-related genes (bar graph of enriched terms colored by *p*-values). (**H**) Summary of the effect of PBX4 mRNA expression on pathway activity across TCGA cancers.

To study the PBX4 molecular mechanism in tumorigenesis and development, we obtained PBX4-related genes to perform functional pan-cancer enrichment analysis. Firstly, the 34 PBX4-binding proteins were screened from the STRING database, and their protein–protein interaction network was constructed as shown in Figure 10A. With the GEPIA2 database, we acquired the top 100 genes strongly correlated with PBX4 expression. Figure 10B showed that PBX4 was positively correlated with LPAR2 (R = 0.45), MUTYH (R = 0.45), CDK11A (R = 0.43), and SRRT (R = 0.41) (all *p* < 0.001). To further explore the possible biological process and pathways involved in PBX4, we performed a cross analysis of the above two groups and found one common gene: ZNF101 (Figure 10C). Interestingly, the positive correlations between PBX4 expression and the above four genes were mostly found in TCGA cancer types (Figure 10D). Next, we used Metascape to perform GO and KEGG enrichment analysis based on the two groups. The GO results revealed that most of these genes were involved in the cell cycle process, MAPK cascade, ncRNA metabolic process, cellular response to starvation, positive regulation of GTPase activity, nucleic acid phosphodiester bond hydrolysis, and regulation of lipase activity (Figure 10E,G). The KEGG results showed that these genes were primarily enriched in cholesterol metabolism, base excision repair, herpes simplex virus 1 infection, transcriptional misregulation in cancer, and Epstein–Barr virus infection (Figure 10F). Additionally, we used GCSA to find that PBX4 was correlated with multiple pathways in 32 cancer types. In particular, PBX4 expression might activate apoptosis, the cell cycle, DNA damage, and hormone AR but inhibited TSC/mTOR, RAS/MAPK, and RTK (Figure 10H).

### 3.9. Pan-Cancer Immune Infiltration Analysis of PBX4

**Figure 11:**
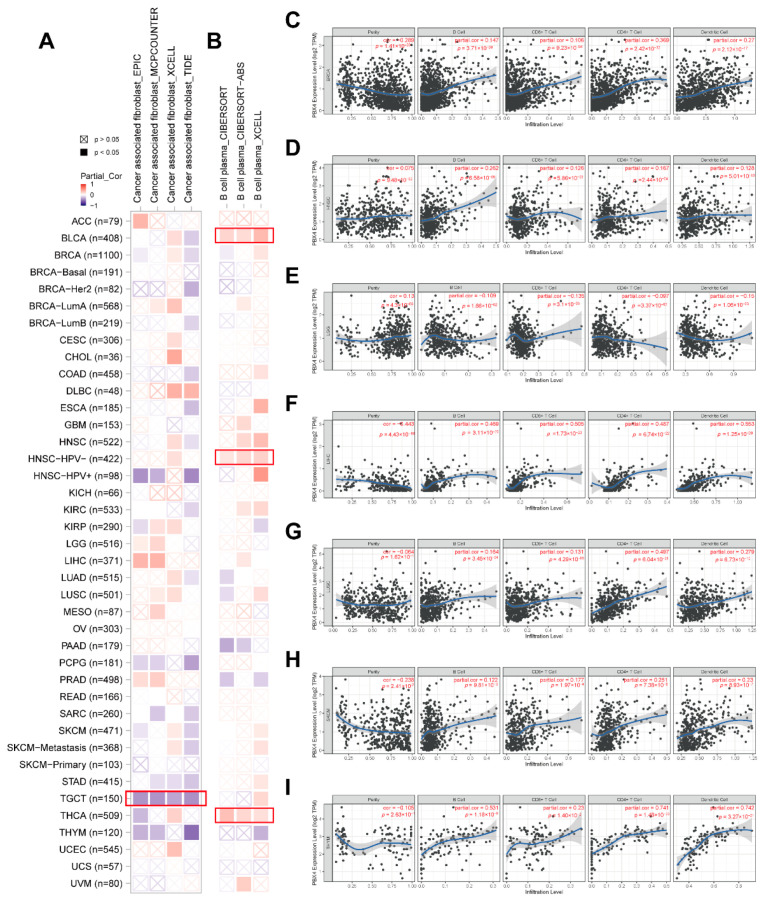
Correlation of PBX4 expression with immune infiltration levels across TCGA cancers. (**A**) Association between PBX4 expression and cancer associated fibroblasts and (**B**) B-cell plasma in different TCGA cancers. Association between PBX4 expression and B cells, CD8+ T cells, CD4+ T cells, and dendritic cells in (**C**) BRCA, (**D**) HNSC, (**E**) LGG, (**F**) LIHC, (**G**) LUSC, (**H**) SKCM, and (**I**) THYM.

Recent studies have revealed that tumor-infiltrating immune cells in the tumor microenvironment are crucial in the initiation, progression, and metastasis of human cancers, and cancer associated fibroblasts might regulate the functions of infiltrating immune cells [39,40]. Based on the RNA single-cell-type specificity, PBX4 was most expressed in several immune cells, including B cells, CD8+ T cells, CD4+ T cells, plasma, and dendritic cells (Figure 2D). Thus, we used TIMER 2.0 to analyze the correlation between PBX4 expression and B-cell plasma in various cancers. The results revealed that there was a positive correlation in BLCA, HNSC-HPV-, and THCA (Figure 11B). Interestingly, the TIMER results showed that the significant positive associations of PBX4 expression with the infiltration levels of the endothelial B cells, CD8+ T cells, CD4+ T cells, and dendritic cells were found in six cancer types (BRCA, HNSC, LIHC, LUSC, SKCM, and THYM (Figure 11C–I), while PBX4 expression was negatively related to the above four immune cells in LGG (Figure 11E) (all *p* < 0.05). With EPIC, MCPCOUNTER, XCELL, and TIDE algorithms, we found that the expression of PBX4 was negatively correlated with cancer associated fibroblasts in TGCT (Figure 11A) (all *p* < 0.05).

### 3.10. Therapy Analysis of PBX4 across Types of Human Cancer

**Figure 12:**
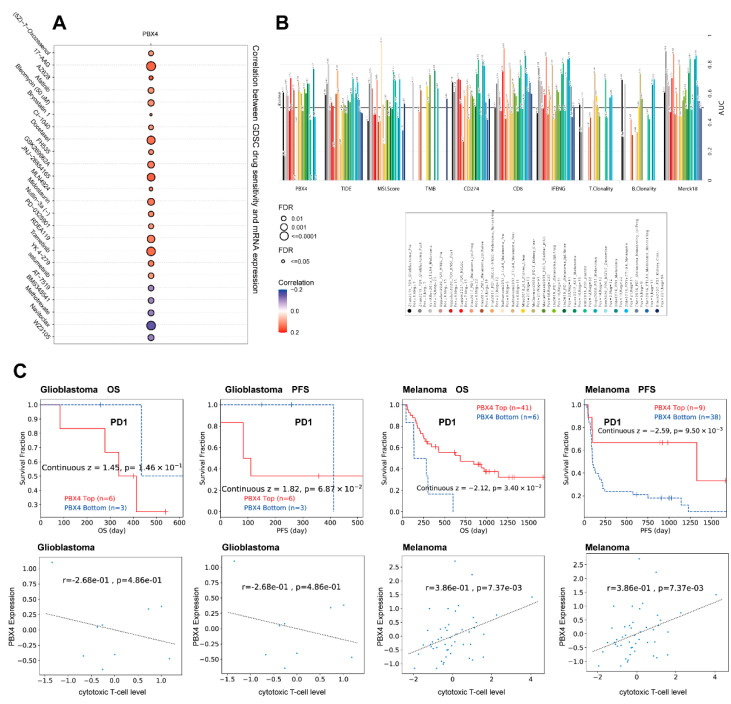
Therapy analysis of PBX4 in different cancer types. (**A**) Drug sensitivity analysis of PBX4 expression in the GDSC database. (**B**) The difference between PBX4 and existing biomarkers in immune checkpoint blockade. (**C**) Correlation between PBX4 expression with cytotoxic T-cell levels and survival curve of immunotherapeutic response.

Next, we analyzed the drug sensitivity of PBX4 expression in different cancers from the GDSC database. It was found that the high expression of PBX4 was strongly positively associated with 50% inhibitory concentration (IC50) values of most drugs except for AT-7519, BMS345541, methotrexate, navitoclax, and WZ3105 (Figure 12A). Furthermore, we used TIDE to find that PBX4 obtained higher predictive values (AUC > 0.5) in 13 of the 25 ICB subcohorts and had more counts of the predictive score than three existing biomarkers (tumor mutational burden (TMB), T-cell clonality (T.Clonality), and B-cell clonality (B.Clonality)) (Figure 12B). As shown in Figure 12C, the PBX4 expression of glioblastoma was negatively associated with cytotoxic T-cell levels and implied some interactions with T-cell exclusion. Moreover, glioblastoma patients with PBX4 low expression could achieve clinical benefits with ICB therapy (programmed cell death protein-1 (PD-1)) and prolong survival durations. On the contrary, low PBX4 expression in melanoma was positively correlated with cytotoxic T-cell levels and suggested a poor prognosis.

## 4. Discussion

PBX4 belongs to the PBX family and acts as a transcriptional cofactor of HOX proteins [41,42], playing critical roles in multiple pathophysiological processes, such as organogenesis [8,9], cell differentiation [11,43], embryonic development [10], and spermatogenesis [13]. Recent studies have revealed that PBX4 involved cancer pathogenesis and progression [44]. However, previous research has mainly studied certain types of cancers. For the first time, we performed an integrative analysis of the roles of PBX4 across TCGA cancers.

Thus, with TCGA, GEO, HPA, and GTEx datasets, we first explored PBX4 expression across various human cancers. The results showed that PBX4 was generally expressed in normal human tissues, especially in the thyroid gland and testis. Meanwhile, the mRNA expression of PBX4 was higher in tissues of testis cancer, thyroid cancer, colorectal cancer, urothelial cancer, stomach cancer, pancreatic cancer, cervical cancer, endometrial cancer, and ovarian cancer. Recent studies have showed that PBX4 was overexpressed in colorectal cancer and Hodgkin lymphoma [16,19], but underexpressed in acute lymphoblastic leukemia [17]. Compared with normal tissues, we found that the upregulation of PBX4 was significantly expressed in BLCA, CESC, CHOL, COAD, ESCA, HNSC, KIRC, KIRP, LIHC, LUAD, PCPG, READ, STAD, and UCEC but significantly downregulated in PRAD, THCA, and KICH. The protein expression of PBX4 was increased in cervical cancer, colorectal cancer, endometrial cancer, and lung cancer tissues. The significant difference in PBX4 expression could imply distinct underlying functions in different cancer types. We further found that PBX4 expression was significantly associated with OS and RFS, but there were distinct differences in different cancers. For instance, the overexpression of PBX4 could prolong the survival of patients with BLCA, CESC, HNSC, KIRP, LIHC, LUAD, STAD, and THCA but shorten the survival of patients with KIRC and UCEC.

Martino et al. found that the overexpression of PBX4 could markedly increase cell proliferation in HCT116 cells of colorectal cancer [19]. Next, we explored the associations between PBX4 expression and pathological stage and tumor grade to understand the PBX4 mechanism of various cancers. Our results revealed that PBX4 was significantly related to the clinical stage of ACC, KIRC, KIRP, LUAD, OV, PAAD, THCA, and UCEC. Meanwhile, as tumor grade improved, the expression of PBX4 significantly increased in HNSC, KIRC, and LGG. These findings suggested that PBX4 might be correlated with the development of multiple cancers. Furthermore, PBX4 expression was significantly different in molecular subtypes and immune subtypes of different human cancers. In particular, PBX4 was significantly related to both molecular subtypes and immune subtypes in five cancers, namely BRCA, KIRP, LGG, LUSC, STAD, and UCEC. We found that there were significant associations between PBX4 expression and molecular subtypes and immune subtypes of different cancers, which might provide a new entry point for understanding the functions of PBX4 in certain cancers.

The genomic alteration may significantly affect tumorigenesis and development [45,46]. Three non-synonymous variants of PBX4 (Pro7Ser, Gly103Glu, and Thr154Met) were identified in the patients with congenital heart defects [47]. Luo et al. revealed that an SNP (rs16996148 in PBX4) was robustly associated with isolated low high-density lipoprotein cholesterol in the Chinese population [48]. We revealed that genetic alterations of PBX4 generally occurred in human cancers and significantly shortened the pan-cancer DFS and PFS. Meanwhile, PBX4 expression was significantly correlated with CNV of HNSC, KIRC, LUSC, LIHC, STAD, TGCT, SARC, ESCA, BLCA, BRCA, UCEC, LUAD, and OV and worsened both the OS and PFS survival time of ACC, CESC, KICH, KIRC, SARC, and UCEC. Kobayashi et al. found that PBX4 was more hypomethylated in naive T helper cells during relapse than in remission of minimal change nephrotic syndrome [49]. He et al. found that PBX4 was related to sarcopenia-related hypermethylation in CpG islands [50]. We found that PBX4 DNA methylation of body and promoter occurred in five tissues: bone, blood, lung, kidney, and liver. Compared with normal tissues, PBX4 was significantly more methylated in BRCA, LIHC, LUAD, LUSC, PRAD, and UCEC but less in BLCA, COAD, HNSC, and KIRP. The MethSurv results revealed that DNA methylation of PBX4 could significantly affect the survival prognosis of various cancers. Moreover, differential methylation levels of PBX4 were correlated with dysfunctional T-cell phenotypes and suggested a survival risk in the brain and head and neck cohorts. These results revealed that PBX4 were not only involved in the epigenetic mechanism of tumorigenesis but also resulted in the immuno-invasive phenotypes and prognosis values of different types of cancer [51].

There were several meaningful findings in our study. For KIRC, we found that overexpression of PBX4 was significantly associated with poor prognosis of OS. Moreover, PBX4 expression was positively related to the clinical stage and the tumor grade. Interestingly, we found that PBX4 expression was significantly correlated with CNV of KIRC and shortened both OS and PFS survival time. There was no study on the role of PBX4 in KIRC. Our findings indicated that PBX4 played an oncogenic role in tumor progression in KIRC and could serve as a biomarker for cancer detection, staging, and prognosis. Molecular experiments could be further needed to validate the oncogenic role of PBX4 in KIRC. For lung cancer, we investigated TCGA-LUAD (*n* = 515) and TCGA-LUSC (*n* = 501) datasets. The results showed that the overexpression of PBX4 was significantly correlated with good OS prognosis and early clinical stage in LUAD but not in LUSC. In addition, PBX4 methylation was negatively correlated with PBX4 expression, and PBX4 hypermethylation of CpG cg11804775 was significantly associated with poor prognosis of patients with LUAD. There is no research on the correlation between PBX4 and LUAD, which may provide a new clue for the study of LUAD.

To further study the potential pan-cancer functions of PBX4, we used CancerSEA, GSCA, and Metascape to perform function analysis. From the single-cell resolution perspective, PBX4 was negatively correlated with DNA repair, invasion, DNA damage, apoptosis, and metastasis in UVM and invasion in BRCA, GBM, and OV. On the contrary, PBX4 was positively associated with stemness in BRCA and apoptosis in OV. Recent studies showed that PBX4 was enriched in the biological processes of cell cycle and cell proliferation in colorectal cancer [19]. We found that PBX4 in most TCGA cancers might participate in the activity of apoptosis, the cell cycle, DNA damage, hormone AR pathways, and the inhibition of TSC/mTOR, RAS/MAPK, and RTK pathways. However, a single gene usually conjoins with other related genes to regulate the biological process of tumorigenesis and progression [52,53]. Thus, we used PBX4-related genes to perform function enrichment analysis. The results showed that they were enriched in the cell cycle process, MAPK cascade, ncRNA metabolic process, cellular response to starvation, positive regulation of GTPase activity, nucleic acid phosphodiester bond hydrolysis, and regulation of lipase activity and mainly participated in the pathways of cholesterol metabolism, base excision repair, herpes simplex virus 1 infection, transcriptional misregulation in cancer, and Epstein–Barr virus infection.

Previous studies showed that infiltrating immune cells could significantly affect tumorigenesis and development, and cancer-associated fibroblasts might regulate infiltrating immune cells [39,54,55,56]. As shown in Figure 2D, PBX4 was mainly expressed in several immune cells. Therefore, we performed immune infiltration analysis to study the critical role of PBX4 in the occurrence and progression of cancers. The results showed that PBX4 expression was significantly correlated with these immune cells in multiple cancer types. Especially in HNSC, we found that PBX4 expression was positively correlated with endothelial B cells, CD8+ T cells, CD4+ T cells, dendritic cells, and B-cell plasma. Meanwhile, high expression of PBX4 was significantly associated with good OS prognosis and high tumor grade. PBX4 expression was negatively correlated with methylation levels but positively with CNV. Furthermore, PBX4 hypermethylation of CpG cg10738025 in HNSC could shorten the survival time of patients with HNSC, and the CNV of PBX4 was significantly correlated with poor OS of HNSC. These results suggest that PBX4 might play a vital role in HNSC. In addition, the TGCT results showed that PBX4 expression was significantly associated with cancer associated fibroblasts in all four algorithms, which implies that PBX4 might affect its transformation and activation.

In addition, we found that PBX4 had more counts of the predictive score than three standardized biomarkers of cancer immune evasion (TMB, T.Clonality, and B.Clonality) in the 25 ICB subcohorts. Rosales et al. found PBX4 mRNA expression increased in etoposide-treated CEM cells, which might be correlated with a more resistant phenotype [17]. Irvin et al. revealed that PBX4 significantly affected low-density lipoprotein cholesterol response to fenofibrate [57]. Intriguingly, we found that high expression of PBX4 was positively related to IC50 values of most drugs except for AT-7519, BMS345541, methotrexate, navitoclax, and WZ3105 from the GDSC database. ICB with targeting PD-1 showed potential antitumor effects for cancer immunotherapy [58,59]. Our findings revealed that the high expression of PBX4 in glioblastoma was correlated with a worse outcome for PD1 immunotherapy, while melanoma patients with a high expression of PBX4 could obtain clinical benefits from ICB therapy and prolong survival time. Thus, PBX4 might serve as an immunotherapy target for various cancers. Additional clinical experiments are needed to further validate our findings.

## 5. Conclusions

In conclusion, our study involved integrative pan-cancer analysis of PBX4. The results showed that PBX4 expression was significantly associated with survival prognosis, molecular and immune subtypes, mutation status, DNA methylation, immune infiltration, and therapy responses in various human cancers, which contributed to understanding the potential roles of PBX4 in various human cancers from several different perspectives. There are several limitations in our study. Firstly, we performed an integrative analysis to investigate the pan-cancer roles of PBX4 with multiple public databases while lacking actual clinical data. Thus, additional clinical data are needed to further verify our findings. Secondly, the specific function and mechanism of PBX4 in the tumorigenesis and progression needs to be further studied with biological experiments.

## Figures and Tables

**Table 1:** The abbreviations and corresponding full names of various cancers.

Abbreviation	Full Name
ACC	Adrenocortical carcinoma
BLCA	Bladder urothelial carcinoma
BRCA	Breast invasive carcinoma
CESC	Cervical squamous cell carcinoma and endocervical adenocarcinoma
CHOL	Cholangio carcinoma
COAD	Colon adenocarcinoma
DLBC	Lymphoid neoplasm diffuse large B-cell lymphoma
ESCA	Esophageal carcinoma
GBM	Glioblastoma multiforme
HNSC	Head and neck squamous cell carcinoma
KICH	Kidney chromophobe
KIRC	Kidney renal clear cell carcinoma
KIRP	Kidney renal papillary cell carcinoma
LAML	Acute myeloid leukemia
LGG	Brain lower grade glioma
LIHC	Liver hepatocellular carcinoma
LUAD	Lung adenocarcinoma
LUSC	Lung squamous cell carcinoma
MESO	Mesothelioma
OV	Ovarian serous cystadenocarcinoma
PAAD	Pancreatic adenocarcinoma
PCPG	Pheochromocytoma and paraganglioma
PRAD	Prostate adenocarcinoma
READ	Rectum adenocarcinoma
SARC	Sarcoma
SKCM	Skin cutaneous melanoma
STAD	Stomach adenocarcinoma
TGCT	Testicular germ cell tumors
THCA	Thyroid carcinoma
THYM	Thymoma
UCEC	Uterine corpus endometrial carcinoma
UCS	Uterine carcinosarcoma
UVM	Uveal melanoma

## Data Availability

The data used to support the findings of this study are available from the corresponding authors upon request.
